# Epstein Barr Virus and Autoimmune Responses in Systemic Lupus Erythematosus

**DOI:** 10.3389/fimmu.2020.623944

**Published:** 2021-02-03

**Authors:** Neelakshi R. Jog, Judith A. James

**Affiliations:** ^1^ Arthritis and Clinical Immunology, Oklahoma Medical Research Foundation, Oklahoma City, OK, United States; ^2^ Departments of Medicine, Pathology, Microbiology & Immunology, University of Oklahoma Health Sciences Center, Oklahoma City, OK, United States

**Keywords:** Epstein-Barr virus, systemic lupus erythematosus, inflammation, autoimmune disease, viral homologs of host genes, molecular mimicry

## Abstract

Systemic lupus erythematosus (SLE) is a complex systemic autoimmune disease. Infections or infectious reactivation are potential triggers for initiation of autoimmunity and for SLE flares. Epstein-Barr virus (EBV) is gamma herpes virus that has been associated with several autoimmune diseases such as SLE, multiple sclerosis, Sjogren’s syndrome, and systemic sclerosis. In this review, we will discuss the recent advances regarding how EBV may contribute to immune dysregulation, and how these mechanisms may relate to SLE disease progression.

## Introduction

Systemic lupus erythematosus (SLE) is a multifaceted systemic autoimmune disease (1) stemming from immune dysregulation. A characteristic feature is the presence of autoantibodies directed towards nuclear antigens (ANA), which can be detected up to a decade before disease onset. Although not completely characterized, studies suggest that cellular dysfunction, dysregulated inflammatory responses and autoantibody -mediated damage leads to progression of autoimmune disease and organ damage (2).

The underlying factors responsible for disease transition and pathogenesis likely involve an interplay between genetic and environmental factors. SLE has a twin concordance rate of 24% to 40% (3, 4) and over 100 genetic associations have been identified and confirmed (5).

Infections or pathogens have been proposed to lead to autoimmunity. Epstein Barr virus (EBV) is one such pathogen that has been repeatedly associated with SLE since the first report in 1969. EBV adopts several strategies to exploit host immune response for its persistence. Consequences to the host are increased acute inflammation and autoantibody generation, which are usually transient and self-limited, as seen in patients with infectious mononucleosis (6). However, a growing body of research suggests that these effects in certain individuals, possibly based on genetic risk factors, can cascade into a chronic inflammatory state. Due to its strong association with tumorigenesis, EBV has been studied extensively for its ability to overcome immune surveillance and approached to combat tumorigenic effects.

**Figure 1:**
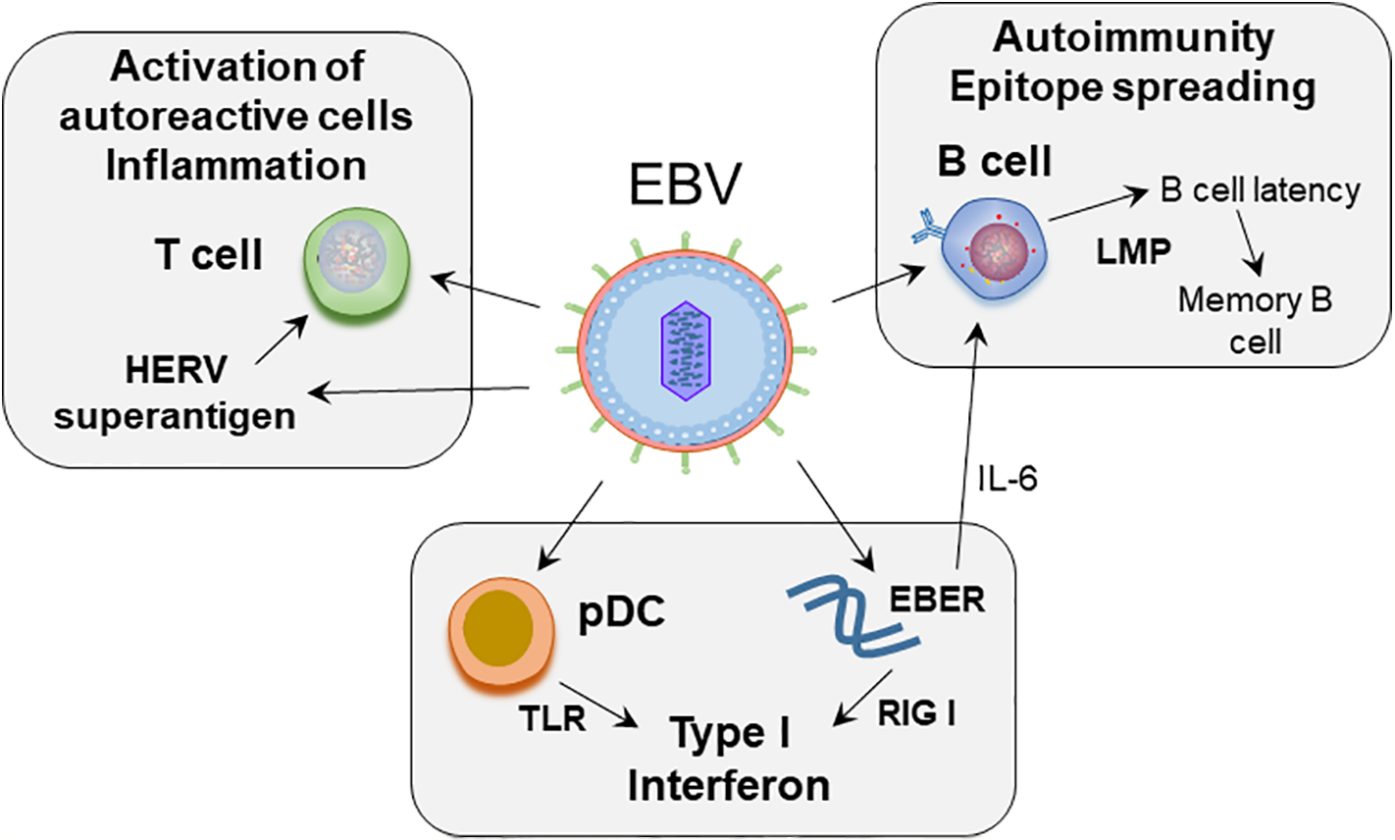
Proposed role of EBV in SLE pathogenesis. EBV infects naïve B cells. The infected B cells enter the memory B cell compartment through germinal center like reaction, mediated by the expression of latent membrane proteins. EBV maintains latency in the resting memory B cells. EBERs, non-coding RNA expressed by EBV, can mimic dsRNA, and activate RIG-I leading to production of type I interferons. EBERs also induce growth factor IL-6 and regulate B cell survival. EBV can act on plasmacytoid dendritic cells (pDC). Initial binding of virus is mediated by class II MHC on pDCs, following which through engagement of TLR7 and 9, EBV RNA and DNA can induce type interferon secretion by pDCs. EBV induces superantigen on HERV-K18, which can induce unregulated T cell activation.

In this review we provide a compilation of the current understanding of how EBV may contribute to immune dysregulation, including strategies used by EBV to combat immune surveillance, and how these processes may relate to SLE pathogenesis (**Figure 1**).

## EBV Life Cycle

Acute primary EBV infection, which is also a common cause of infectious mononucleosis, is characterized by fatigue, atypical lymphocytosis, splenomegaly, and lymphadenopathy. Although the host immune response eventually controls viremia, the virus maintains latency in memory B cells with occasional reactivation to infect naïve B cells. EBV genomes in latently infected B cells are thought to exist as episomes (7), although it is possible that the genomes exist as integrated DNA. EBV expresses nine latent proteins; six EBV nuclear antigens (EBNA, EBNA-1, 2, 3A, 3B, 3C, and leader protein), and three latent membrane proteins (LMP 1, 2A, and 2B). In addition to latent proteins, expression of small non-polyadenylated RNAs, EBER1 and 2, is also observed (8).

Unique forms of latency that differ in the latent protein expression have been identified (**Table 1**). Latency III, where all latency gene products are expressed, is the predominant latency observed in lymphoblastoid cell lines, acute infectious mononucleosis, and certain immunocompromised individuals. This form of latency can mediate naïve B cell activation. EBNA1 and LMP1/2A are expressed in the latency II program, which is observed in nasopharyngeal carcinoma and Hodgkin’s lymphoma. LMP1 and LMP2 can induce B-cell activation and growth (proliferation). Latency I, which is observed in EBV-positive Burkitt’s lymphoma tumors, expresses only EBNA-1. In this form, latent EBV genomes can multiply in dividing memory cells. The Gly-Ala repeats in EBNA-1 inhibit antigen processing, and therefore, CD8 T cells are unable to detect virally infected cells in this form. Latency 0 is observed in quiescent B cells, where no EBV proteins are expressed, however cells switch to Latency I during cell division with expression of EBNA-1, which is required for replication of the episome. Latently infected B cells occasionally reactivate EBV. This allows the virus to re-infect new B cells and epithelial cells, and acts as a source of viral transmission. Although the molecular pathways involved in viral reactivation are studied extensively, the triggers for reactivation *in vivo* are unclear.

**Table 1:** Latency forms of EBV.

Latency	Genes expressed
Latency 0	EBER1/2
Latency I	EBNA-1, EBER1/2, miRNA
Latency II	EBNA-1, EBER1/2, miRNA, LMP1/2
Latency III	EBNA, EBNA-1, 2, 3A, 3B, 3C, EBNA-LP, LMP 1, 2A, 2B, EBER1/2, miRNA

The occasional reactivation of the virus can be detected serologically. A primary infection with EBV leads to an IgG response to viral capsid antigen (VCA). The VCA IgG antibodies are maintained throughout the life span of the individual. Following VCA IgG response, IgG responses toward early antigen (EA) are detected. These antibodies are detectable for 6 months to up to two years. During EBV reactivation, EA IgG levels are detectable and there is an increase in VCA IgG levels (9). Therefore, an increase in VCA IgG and presence of EA IgG indicates current or recent reactivation of the virus.

## EBV Latency and Reactivation in SLE

Many studies to date have demonstrated an association between SLE and EBV infection. A higher EBV seroconversion rate was observed in both pediatric and adult SLE patients compared to healthy controls (10–12). SLE patients show increased levels of IgG antibodies toward VCA and EA, both indirect markers of increased EBV reactivation. However, the IgG responses towards other herpes viruses such as cytomegalovirus (CMV) and herpes simplex virus (HSV) are similar in SLE patients and controls. These reports suggest that SLE patients may have increased reactivation of the virus. EBV viral load is elevated in SLE patients (13, 14), which may also suggest increased reactivation. A possible reason for increased reactivation is inefficient regulation of the latent phase or enhanced transition from latent to lytic phase. Interestingly, a higher percentage of patients have detectable levels of EBV gene BZLF1 (15), which is an immediate-early gene that is responsible for the switch to lytic cycle. Two other latent genes LMP1 and LMP2A were also detected in SLE patients. The type of latency maintained in SLE patients is not completely understood. LMP1/2A are expressed in latency II, and all latent genes are expressed in latency III (**Table 1**). The presence of two latent genes, BZLF-1 and LMP-1, which cannot be detected in seropositive healthy individuals, suggests that EBV latency may be dysregulated in some SLE patients. Based on the expression pattern of latent genes reported so far, SLE patients may have an intermediate form between latency II and III.

## EBV Reactivation in SLE and Underlying Mechanisms

Based on serologic evidence and higher viral loads observed in SLE patients, the consensus is that SLE patients have increased EBV reactivation. Dysregulation of anti-viral T cell responses is a proposed mechanism for increased viral loads. SLE patients have higher interferon γ (IFNγ) secreting CD4+ T cells, but lower frequencies of EBV specific CD8+ T cell responses. EBV viral loads in peripheral blood cells positively correlated with EBV specific and IFNγ secreting CD8+ T cells (14). EBV specific CD8+ T cells in SLE patients are functionally impaired (16, 17), although CMV specific responses were unaltered (17). The upregulation of PD1 on EBV specific T cells in SLE patients may be responsible for the suppressed responses to EBV antigen, as blockade of PD1 restored IFNγ production in response to EBV antigens. Based upon these data and the observed diminished responses of SLE T cells to superantigen stimulation, the authors suggest that SLE T cells demonstrate an exhausted phenotype. However, CMV specific T cell responses were unaltered by PD1 blockade. These data suggest that the general immune surveillance mechanisms are intact in SLE patients, but there is an inherent defect in regulating EBV infection (17). Both CD4 and CD8 lytic and latent antigen specific functional T cells were lower in SLE patients. A negative correlation between SLE disease activity index (SLEDAI) and EBV specific functional T cell responses was reported (18), with decreased EBV lytic gene responsive T cells in patients with elevated disease activity. Furthermore, an inverse relationship was observed between EBV specific T cells and levels of anti-EBV antibodies (18). SLE T cells may also contribute to defective regulation of certain B cell functions (19). Absolute numbers of Th17 and Treg cells were reduced in SLE patients with EBV and/or CMV viremia compared to those without viremia or healthy controls. However, there was a direct correlation between viremia and SLEDAI, suggesting that reduction in Th17 and Treg cells may be a consequence of SLE immune dysregulation independent of viremia (20). EBV can transactivate superantigen on human endogenous retroviral (HERV)-K18, which can lead to unrestricted activation of T cells (21).

EBV induced gene 3 protein (EBI3) was identified in EBV transformed B cells (22). It serves as a beta chain for cytokines IL-27, IL-35, and IL-39, and can induce regulatory or suppressive T cells in a murine model (23). The serum IL-35 level and the percentage of CD4+EBI3+ T cells were negatively correlated with the SLE disease activity index, and both of these parameters were increased shortly after treatment of active SLE patients with methylprednisolone (24). However, levels of EBV reactivation were not determined in this study. Although EBI3 was initially reported in EBV transformed B cells and induced by LMP1, the name of the gene is misleading. EBV infection of T cells is not established unequivocally. It was later shown that EBI3 can be induced in naïve T cells by polyclonal stimulation with plate bound anti-CD3 and anti-CD28 (25). This also explains upregulation of EBI3 in experimental murine models, which lack EBV infection. Therefore, the increase in IL-35 observed in SLE patients may be independent of EBV induced gene expression. Studies evaluating the upregulation of EBI3 in SLE patients in the context of EBV infection and subsequent contribution to SLE pathogenesis are lacking.

Differences in cytokine production in response to EBV antigens have been reported. SLE patients exhibited a decreased IL-12, IFN*γ*, IL-17, and IL-6 response to EBNA-1, and decreased induction of IL-6, TNF*β*, IL-1*β*, and GM-CSF upon EBV-EA-D stimulation. Serologic SLEDAI scores, based solely on anti-dsDNA, complement, thrombocyte, and leukocyte levels, correlated negatively with numerous cytokine responses against EBNA-1 and EA-D (26). These data further support impaired regulation of immune response against latent and lytic EBV antigens in SLE patients.

The numbers of infected B cells positively correlated with SLE disease activity index (15). The EBV viral load in SLE patients with active disease was found to be higher than in inactive cases (27), however, another study did not find this (17). Although this report did not find a consistent increase in EBV viral load immediately prior or at the time of a flare, the viral loads were higher in a majority of patients during elevated disease activity (17), suggesting that EBV may have a role in the pathogenesis and activity of SLE. The overall low number of EBV-infected B cells during latency and the lower numbers of B cells due to lymphopenia in SLE patients provides a technical challenge in detecting EBV DNA. Assays with higher sensitivities to detect both latent and lytic EBV genes, perhaps partnered with single cell technologies, will be helpful to understand the relation between timing of EBV reactivation and SLE flare. Detailed longitudinal analyses of a larger cohort of SLE patients will improve our understanding of viral reactivation and SLE disease activity.

Newer data have evaluated the association of EBV reactivation with SLE disease onset. Our retrospective analyses of unaffected family members of SLE patients showed that SLE relatives that subsequently transition to classified SLE (>4 ACR criteria), have increased VCA-IgG and EA-IgG at a time-point prior to the transition, when compared to relatives that do not transition to SLE (28). The responses towards CMV and HSV-1 were not different between the two groups of relatives. These data suggest that EBV reactivation observed in SLE patients is not due to immune dysregulation caused by the chronic autoimmune and inflammatory environment in patients, nor is it solely a consequence of immunosuppressive medications. However, as the study involved blood relatives of SLE patients, a genetic component may be involved in increased EBV reactivation. On similar lines, seropositivity for anti-EBV early antigen (EA), a marker of EBV reactivation, was dramatically increased in patients with SLE compared with unrelated controls (92.3% vs 40.4%; OR 17.15(95% CI 10.10, 30.66), p<0.0001) or unaffected first-degree relatives of lupus patients (49.4%; OR 12.04(7.42, 20.74), p<0.0001). The seroprevalence of VCA IgG in patients and first-degree relatives was similar suggesting same level of prior EBV exposure in these two groups (29).

Significant interactions between EBV serology and single nucleotide polymorphisms (SNPs) in genes that are associated with SLE and also involved in EBV infection were observed. The association between VCA IgG level and transitioning to SLE was modified by *CD40* rs4810485 (interaction p = 0.0009). Similarly, the association between VCA IgA and transitioning to SLE was modified by *IL10* rs3024493 (interaction p *=* 0.008) (28). In line with a genetic component contributing to increased EBV reactivation, a higher frequency of subjects with germ-line mutations in CTLA-4 had detectable EBV viral load when compared with healthy controls. None of the subjects had symptoms of EBV infection the time of analyses. However, none of the 15 subjects included in this study had a SLE diagnosis (30). Parks et al. showed a significant interaction between VCA IgA and CTLA-4 gene polymorphism (-1661AA), and increased VCA IgA seropositivity in African American SLE patients (31). CTLA-4 -1661 mutation was associated with risk of SLE in young African American patients (32).

Harley et al. recently showed that in EBV-immortalized B cells, almost half of SLE European ancestry risk alleles can be occupied by EBNA-2 protein, which is expressed in latency II and III. The authors showed that host transcriptional factors bind to SLE risk loci only in the presence of EBV, and that EBNA-2 is involved in allele dependent transcription complex formation at risk loci. These data provide another potential origin of gene/environment interaction in SLE (33).

Thus, genetic predisposition leading to immune dysregulation may contribute to EBV reactivation eventually resulting in classified SLE.

## EBV Effects on the Immune System in SLE

In order to evade the host immune system and to establish a persistent latent infection, EBV encodes several viral homologues of human proteins. These homologues either accentuate the effect of human proteins on immune cells, inhibit, or allow the virus to hijack the immune response to its benefit.

### EBV IL-10

EBV IL-10 (vIL-10) is a late viral gene expressed during the lytic phase of virus replication encoded by the viral BCRF1 gene, which is highly homologous to the human IL-10 (hIL-10) gene (34, 35). Due to the high homology, vIL-10 shares some of the suppressive and stimulatory functions of hIL-10. vIL-10 can inhibit inflammatory cytokine (IFNγ, TNFα) production and can promote proliferation and differentiation of B cells, as well as immunoglobulin production. Functional differences between hIL-10 and vIL-10 have also been reported. vIL-10 cannot co-stimulate mouse thymocyte proliferation and mast cell proliferation and cannot up-regulate MHC class II on B cells (36–38).

We recently showed that in contrast to hIL-10, vIL-10 can induce a pro-inflammatory phenotype in monocytes. vIL-10 induced a unique gene expression profile in monocytes, and monocytes exposed to vIL-10 showed defective clearance of apoptotic cells. vIL-10 signals through the same receptor subunit as hIL-10, can act as a competitive inhibitor of hIL-10, and inhibit suppressors of immune response induced by hIL-10. vIL-10 levels were significantly higher in SLE patients plasma compared to matched controls (39). As vIL-10 is a lytic gene, these data also support increased reactivation of EBV in SLE patients.

**Figure 2:**
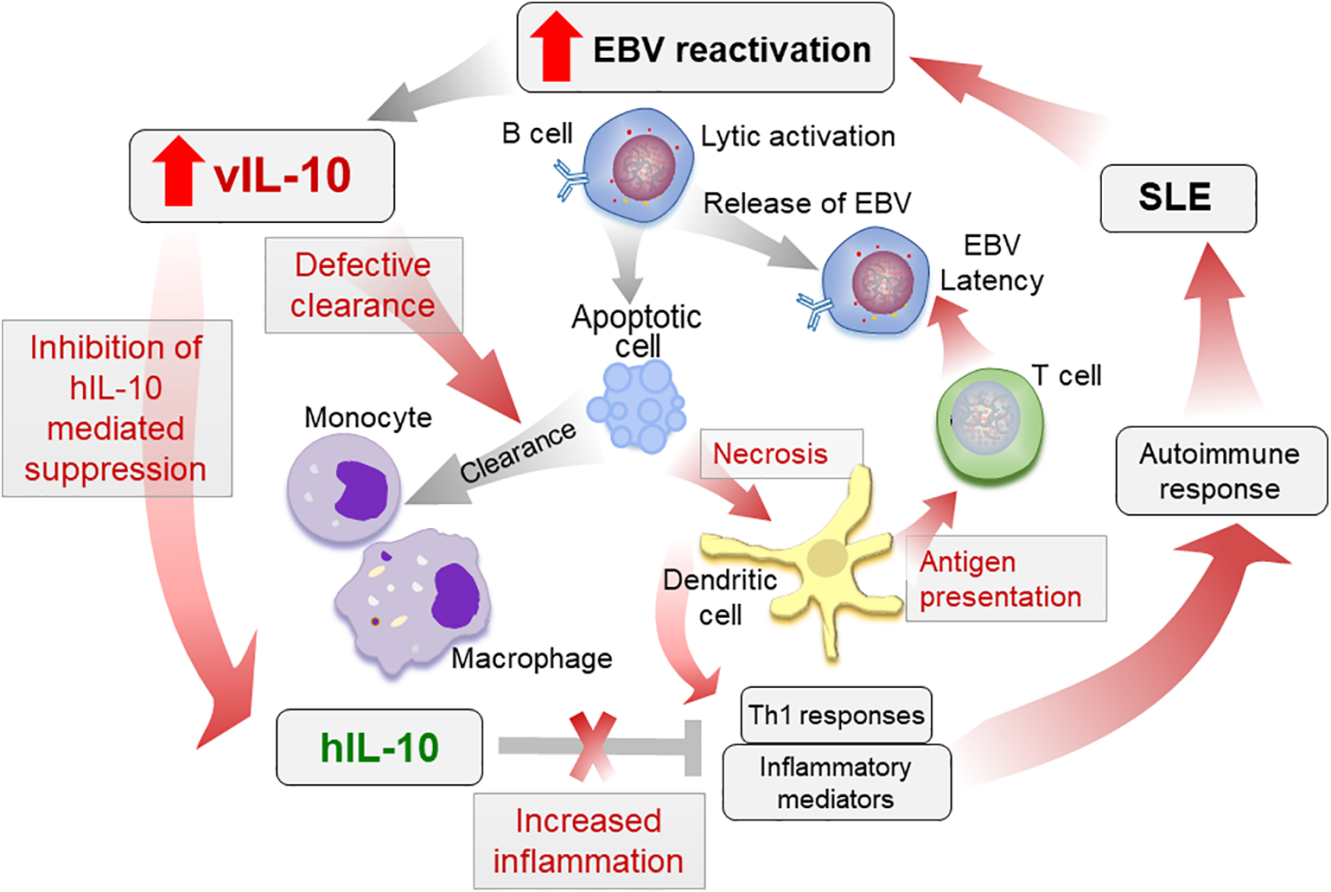
Proposed role of vIL-10 in SLE pathogenesis. Increased reactivation of EBV in SLE patients increases vIL-10. vIL-10 competes with hIL-10 for IL10R1 and inhibits the suppressive effects of hIL-10 on myeloid cells. vIL-10 also reduces the ability of monocytes/macrophages to clear apoptotic cells. This leads to increased secondary necrosis, increased presentation of antigens by dendritic cells (DCs), and allows virus to establish latency. Reduced clearance of apoptotic cells leading to secondary necrosis along with increased antigen presentation and inflammatory responses exacerbate autoimmune response in SLE. The processes possibly regulated by vIL-10 are shown in red arrows.

Increased vIL-10 in SLE patients may increase pro-inflammatory responses by monocytic cells, while inhibiting hIL-10 functions. These pro-inflammatory mediators, along with a reduced clearance of apoptotic infected cells, may lead to increased antigen presentation and activation of cytotoxic T cell responses towards EBV. Indeed Stewart et al. showed that vIL-10 enhances the generation of allo-specific CTL, EBV-specific CTL, and HLA-unrestricted activated killer cells (40). Although this allows the virus to enter latency and persist, in a genetically prone individual with defective tolerance checkpoints, the defective clearance and increased antigen presentation may lead to autoimmune responses (**Figure 2**). Further longitudinal studies evaluating vIL-10 levels in preclinical samples, as well as in SLE patients, pre and post flare, and associations of these levels with monocyte activation status are needed to confirm the role of vIL-10 in induction of an autoimmune response.

How vIL-10 induces a unique gene expression in monocytes and can inhibit hIL-10-mediated immune suppression is not clear. vIL-10 has lower affinity to IL-10R1 compared to hIL-10. However, vIL-10 is more potent than hIL-10 in inducing B cell proliferation, and therefore the lower affinity may not explain the differences in monocyte activation by vIL-10. The vIL-10: IL-10R1 interaction is very transient, while with hIL-10 is more sustained (41). A transient interaction may interfere with ligand-dependent receptor internalization and proteasomal degradation. vIL-10 may be sequestering receptors and compete with hIL-10. Although not reported in the literature yet, it is possible that the vIL-10 monomer forms a heterodimer with hIL-10 and inhibits signaling by hIL-10.

### Latent Membrane Proteins

How EBV maintains latency in memory B cells is also not completely understood. It is hypothesized that EBV enters the memory B cell compartment through differentiation of the latently infected B cell blasts into resting memory B cells, also known as the germinal center (GC) model. The observations that the viral infection is strictly latent in resting memory B cells in the periphery, but active infection of naïve B cells and virus shedding can be detected in tonsillar lymphoid tissue, support this hypothesis [Reviewed in (42)].

EBV expresses three latent membrane proteins (LMP, 1, 2A, 2B) that can mimic signals necessary to rescue normal B cell differentiation in absence of T cell signals. Despite the lack of significant protein homology, LMP1 is a functional homologue of CD40, and acts as a constitutively active receptor (43). LMP1 induces B cells to express B cell-activating factor of the TNF family (BAFF) and a proliferation-inducing ligand (APRIL), which mediate B cell survival and T cell-independent antibody production, and therefore can induce class switch recombination (CSR) in absence of a GC reaction (44, 45). Thus, EBV may block B cells from entering GC, and induce extra-follicular B cell activation through the expression of LMP1. The expression of a chimeric molecule with the mouse CD40 extracellular domain and the LMP1 intracellular signaling regions in lupus-prone mouse strain accentuated the autoimmune phenotype. This suggests that LMP1 acts synergistically with host predisposing genetic factors and contributes to exacerbation of an autoimmune response (46).

LMP2A mimics the B cell receptor (BCR), and contains an immunoreceptor tyrosine based activation motif (ITAMs) which associates with downstream signaling kinases. LMP2A mimics the BCR signal and can rescue B cells lacking surface immunonoglobulin from death (47). Conditional expression of LMP2A in murine GC B cells enhanced BCR signals, facilitated plasma cell differentiation, and resulted in selection of low affinity antibody producing cells. The conditional GC expression also led to SLE-like autoimmune phenotype including anti-double stranded DNA (dsDNA) antibody production, and immune complex deposition in the kidneys (48). Expression of LMP2A transgene in anti-Sm heavy chain transgenic mice resulted in increased anti-Sm antibodies (49). In these mice transgenic for anti-Sm and LMP2A, anti-Sm B cells bypassed the pre-plasma cell tolerance checkpoint and differentiated into antibody secreting cells, suggesting that LMP2A can modify GC B-cell selection and may contribute to persistent EBV infection.

### EBV RNA (EBERs and miRNA)

Additional genes that are expressed during EBV latency are two noncoding RNAs, EBER1 and EBER2, and 44 microRNAs (miRNAs), derived from two loci, the BART and BHRF clusters. BART transcript encodes 22 miRNA precursors (miR-BART1-22) with 40 mature miRNAs, whereas the BHRF1 transcript expresses three miRNA precursors (miR-BHRF1-1, -2, and -3) producing four mature miRNAs (50). EBV miRNA from infected cells were secreted in exosomes, which can be internalized by monocyte derived dendritic cells (51) and modulate their gene expression. In individuals with increased EBV viral load, EBV miRNA were detected in both B and non-B cells in peripheral blood. Although the levels of EBV miRNA have not been compared between SLE patients and unaffected donors, EBV miRNA may be contributing to differences in gene expression profiles observed in non-B cells in SLE patients.

EBER1 and EBER2 are present in all four latency stages (52, 53). Several reports have suggested a role for EBERs in the tumorigenic process *in vivo*, which are also supported by murine studies where transgenic mice expressing EBER1 developed lymphoid hyperplasia and an increase in lymphoma incidence (54). EBERs form a stem–loop structure by intramolecular base-pairing, which can give rise to dsRNA-like molecules (55, 56). EBERs can bind to dsRNA activated protein kinase PKR, inhibit its phosphorylation and can confer resistance to IFN-induced apoptosis in Burkitt’s lymphoma cells (57). EBERs can contribute to B cell transformation and growth by inducing the growth factor IL-6 (58). EBERs can regulate target regulation of several miRNAs. Expression of EBER can enhance the inhibitory effect of miR143-mediated downregulation of the inflammatory gene IL1α (59), however, the significance of these effects in the development or progression of autoimmune diseases is unclear. Expression of EBER in EBV-negative B lymphoma cell line resulted in upregulation of kinases involved in B cell pro-survival signaling, which were previously considered to be regulated solely by LMP1, suggesting a redundancy in function between EBERs and LMP1 during latency (60). EBERs are recognized by retinoic acid-inducible gene I (RIG-I) through the helicase domain and can activate signaling to induce type I interferon and interferon-stimulated genes (61).

SLE patients show increased levels of type I interferon in serum, and SLE disease activity correlates with IFNα levels and the strength of the interferon signature (62, 63). EBV increases IFNα secretion by plasmacytoid dendritic cells (pDCs) through toll-like receptors (TLR). The recognition of EBV is mediated by class II MHC molecules (64). The increased LMP1 gene expression in SLE patients correlated with SLE disease activity index (SLEDAI) and interferon induced gene expression (65). The levels of EBERs were not evaluated in this study. The contribution of EBV or EBER mediated interferon activation and the significance of this induction in progression of SLE needs further evaluation.

## EBV and Autoimmune Humoral Response in SLE

In SLE patients, EBV EA IgG positivity correlated with lupus antibodies (29). EBV IgG also correlated with anti-Ro and anti-La antibodies in SLE patients (66).

Molecular mimicry between SLE autoantigens and EBV antigens may lead to autoimmune response. Antibodies towards different regions of EBNA-1 protein cross-react with SLE autoantigens SmB, SmD, as well as Ro (67). Monoclonal antibodies generated from mice immunized with EBNA-1 cross-react with dsDNA (68, 69). Cross-reactivity between the anti-EBNA-1 response and anti- complement component C1q response has also been shown. Anti-C1q antibody towards A08 epitope of C1q isolated from SLE patients can bind a peptide derived from EBNA-1, EBNA348, and SLE patients that showed reactivity to EBNA348 peptide had higher levels of anti-C1q. This cross-reactivity was shown to be dependent on amino acid identity (70). Peptides derived from EBV EA and LMP1 increased ANA positivity in mice. Both these peptides increased anti-SmB and anti-SmE. While EA derived peptide, EP4, additionally increased anti-SmD and anti-Ro, LMP1 derived peptide increased anti-rRNP. Levels of EP4 antibodies were higher in SLE patients and correlated with SLEDAI. Interestingly, both these peptides had about ~60% amino-acid sequence similarities with self-peptides, but the percentage of similarities with amino-acid characteristics was 75 and 70% respectively for each peptide (71).

Immunization of experimental animals with peptides from regions of EBNA-1 lead to lupus-like autoimmune disease (72–74). In these studies, immunization with a single peptide lead to the generation of cross-reactive antibodies, but the autoimmune response also spread to several different epitopes, and was not restricted to the cross-reactive epitope. Furthermore, injection of mice with plasmids expressing either full-length EBNA-1 or EBNA-1 lacking 15 amino acids in in the Gly-Ala repeats, resulted in anti Sm, and anti-dsDNA antibodies (75). Epitope spreading has been suggested as a possible mechanism for accrual of antibody specificities, and has been shown to occur with immune response towards spliceosomal and other proteins (72, 74, 76).

**Figure 3:**
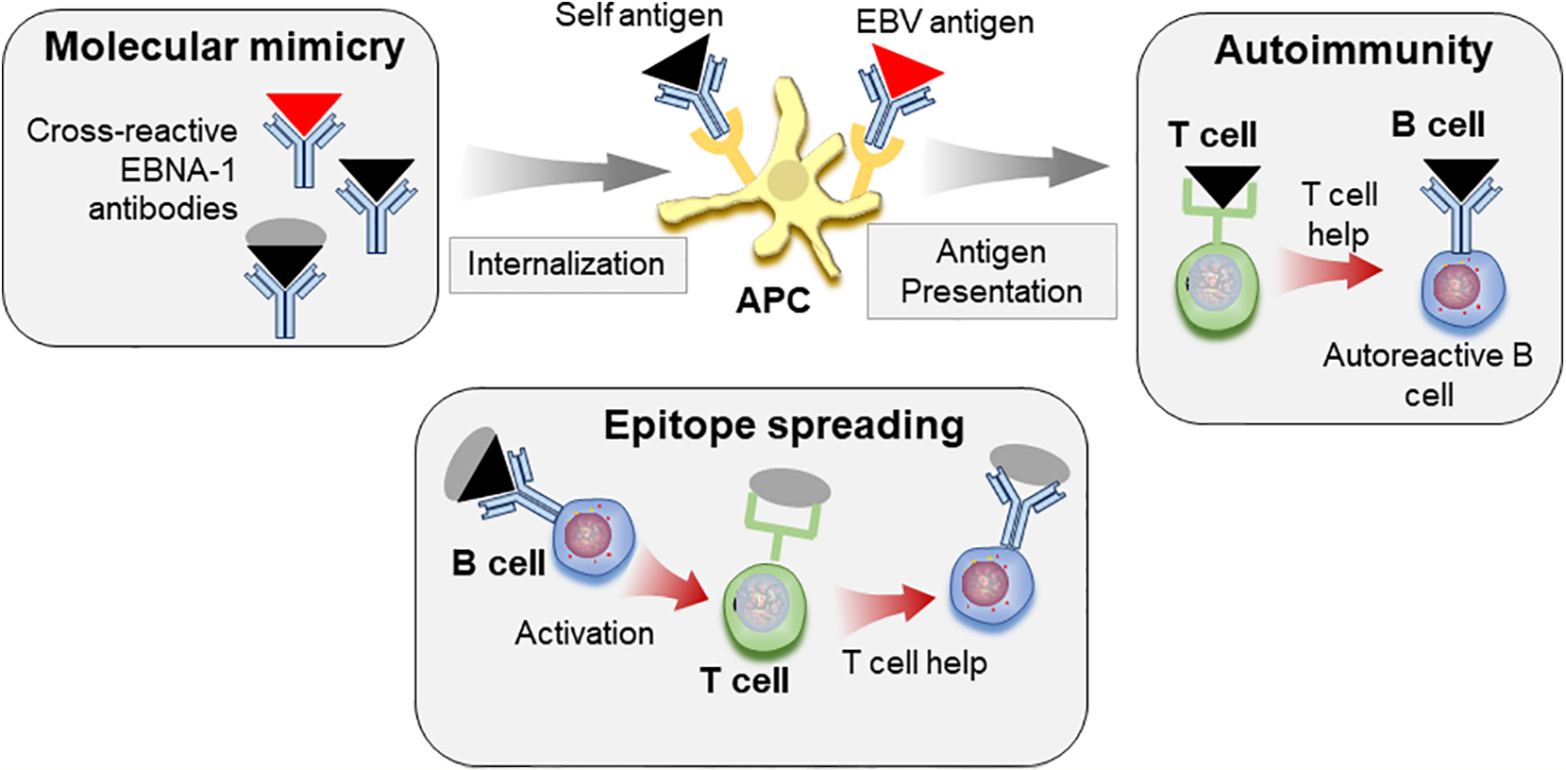
Molecular mimicry and epitope spreading in autoimmunity. Antibodies to viral antigens such as EBNA-1 (red triangle) cross-react with autoantigens (black triangle) due to structural similarities. Immune complexes consisting of autoantigen/antibody complexes are internalized by antigen presenting cells (APC), antigen is processed and peptides presented to T cells, which can allow for loss of tolerance. Defective T cell tolerance possibly contributed by genetic susceptibility may be responsible for this loss of tolerance. These autoreactive T cells in turn provide help to auto-reactive B cells leading to autoreactive antibody response. The self-protein bound to B cell receptor is internalized, processed, and presented to T cells. The autoimmune response can be further diversified by epitope spreading. B cells specific for viral antigen (red triangle) can recognize similar structures on self-antigen (black triangle). However, these cells can internalize and present peptides from whole protein that carries the cross-reactive epitope (black triangle+gray circle) to T cells, which then provide help for antibody response towards additional epitopes on the protein by B cells.

Taken together, these reports suggest that molecular mimicry with EBV epitopes may allow loss of tolerance to self-antigens. Through the process of epitope spreading, these responses may target additional self-epitopes, eventually leading to pathogenic responses and to clinical SLE (**Figure 3**).

## Animal Models of EBV Infection

Although a significant effort has been made to understand EBV biology, understanding how EBV contributes to autoimmune pathogenesis, and the causal relationship between EBV infection, reactivation and autoimmunity are limited due to lack of an appropriate animal model. Peptide immunizations have been instrumental in establishing molecular mimicry between EBV antigens and autoantigens. Transgenic mouse model approaches allowed a better understanding of the ability of EBV latent proteins to modulate B cell function. However, the expression of EBV encoded oncogenes in absence of the entire EBV genome has limitations. These knowledge gaps warrant a suitable animal model that recapitulates the features of EBV infection.

Non-human primates are infected naturally with EBV-related herpesviruses, or lymphocryptoviruses (LCV), and are therefore considered as models for EBV infection [reviewed in (77)]. A primary EBV infection can be established in healthy New Zealand white rabbits, and EBV can also infect Owl monkey and marmosets (78–80). These animal models may prove to be useful for understanding role of EBV in malignancies. However, none of these are characterized as animal models for human autoimmune diseases.

A major advance in establishing a mouse model for EBV came from utilization of humanized models on an immune-deficient murine background. The reconstitution of severe combined immune-deficient (SCID) mice with human peripheral blood leukocytes results in mice with inducible human immune function (81) and development of EBV+ lymphomas by transfer of peripheral blood leukocytes from EBV positive donors (82). However, several limitations such as transient nature of the graft, low engraftment levels, and frequent graft-versus-host disease caused by human T cells attacking mouse tissues, limit the use of this model.

Reconstitution of recombination activating gene 2 (Rag2) deficient IL2 receptor gamma (IL2Rγ) deficient mice also supported EBV infection (83). The deficiency of IL2Rγ allows for T cell re-constitution, and T cells are selected on murine tissue. However, as the T cell are selected on murine and not human tissue, the response in these mice is still suboptimal. This limitation can be overcome by implanting Non-obese diabetic (NOD)/SCID mice with human fetal liver and thymic tissue to provide human T cells appropriate thymic environment, with subsequent autologous CD34+ cell implantation following sub-lethal irradiation (BLT mice) (84). BLT mice showed marked increase in memory T cells, and the T cells could respond to autologous antigen presenting cells upon EBV infection, suggesting that human T cells in BLT mice can mount human-MHC-restricted response and can be used to reproduce human T and B cell interactions. Although an attractive approach, humanized models of EBV infection have not been utilized for SLE research yet. Reconstitution of immunodeficient mice with hematopoietic stem cells from EBV positive and EBV negative SLE patients and matched controls may provide useful insights into pathways regulating increased reactivation in SLE and/or role of EBV in disease progression.

EBV infection of NOD/SCID IL2Rγ-/- (NSG) mice reconstituted with human cord blood hematopoietic cells resulted in erosive arthritis in 65% of mice (85). However, neither anti-citrullinated peptide antibodies nor rheumatoid factor were detected in the blood of affected mice. The serological response to EBV infection observed in humans was also not detected, suggesting that the arthritis observed in these mice was by mechanisms different from those in patients. However, the genetic factors associated with rheumatoid arthritis were not considered in this study.

The study does point out a possible limitation of using humanized mouse models to replicate EBV infection. During both primary infection and subsequent reactivation, lytic replication of EBV occurs in oropharyngeal epithelial cells, where infectious virus particles are produced and shed. Although EBV is hypothesized to infect and to maintain latency only in B lymphocytes (86), EBV can replicate in epithelial cells and viral gene expression patterns differ when the virus emerges from epithelial cells versus B cells, which suggests passage back and forth (87). Due to differences in routes of infection and lack of the epithelial infection, humanized mice do not recapitulate the complete life cycle of EBV infection, and therefore do not reflect the immune response to EBV infection. These models also lack final lytic replication in oropharyngeal epithelial cells, which the virus uses to amplify infectious virus production during shedding into saliva. This limitation may be overcome by human epithelial tissue grafts in humanized mice followed by infection through the natural route. However, whether the transient infection in epithelial cells that produces virus with increased tropism to B cells is necessary to establish latent EBV infection in B cells and whether this transient infection occurs during EBV reactivation are not known.

A murine virus similar to EBV is an alternate approach. The most probable is murine gamma herpes virus 68 (MHV68). Although not identical to EBV, MHV68 shares several features. MHV68 is found in class switched B cells that have undergone GC reaction and reflect memory B cells. MHV68 is a natural pathogen of free-living murid rodents. Virus neutralizing antibodies are detectable in the natural hosts (88). The infection of mice with MHV72, a gamma herpesvirus strain related to MHV68, leads to detectable anti-viral antibodies, and these correlate with viral reactivation (89).

MHV68 infection is associated with an expansion of lymphocyte populations that drives an infectious mononucleosis-like response marked by enlarged lymph nodes and splenomegaly (90, 91). Productive infection in the lungs following intranasal infection of mice with MHV68 lasts for ~10 days. During this time the virus spreads to spleen through infected B cells and establishes latency in GC B cells (92). Long term latency is detected in IgD- subset of splenic B cells (93). MHV68 has been shown to maintain latency in peritoneal macrophages, which has not been reported for EBV. However, similar to EBV, the splenic latency is solely dependent on B cells (94).

MHV68 increased anti-Sm antibodies in wild type and lupus prone mice during acute phase of infection, however, chronic infection protected mice from lupus-like disease (95). The frequency of infected cells and viral load was not determined, and single high dose of virus was used, which was administered intra-peritoneally. Lower doses of virus do not impact establishment of latency but can delay the acute-phase replication peak. Small numbers of pre-formed virus particles were detected in splenocytes of mice infected with lower doses of the virus (96). Although this small increase in the numbers of virus particles did not constitute significant reactivation in the non-autoimmune wild type C57/Bl6 strain used in that study, it may contribute to immune response in a mouse strain genetically prone to immune dysregulation. Therefore, administration of lower doses of MHV68 to lupus-prone mice by oral and/or intranasal routes, may recapitulate EBV infection in SLE patients. MHV68 does not encode a homologue for human IL-10. However, a recombinant MHV72 expressing EBV IL-10 showed exacerbated acute-phase pathogenicity (97). The effect of this recombinant virus on lupus like disease in murine models has not been evaluated. Detailed analyses of humoral response to MHV68, frequency of viral reactivation, and frequency of infected memory B cells in lupus prone mice are necessary to understand the role of MHV68 in murine lupus-like disease.

## Concluding Remarks

EBV can modulate immune responses in a myriad of pathways, including generation of cross-reactive antibodies, IFNα secretion, antigen independent B cell activation, gene expression modification, and anti-inflammatory response suppression. SLE patients show evidence of increased reactivation of EBV, possibly resulting from dysregulated immune responses together with genetic risk factors. Furthermore, the viral homologues such as vIL-10 modulate immune response in a manner that can exacerbate autoimmune response in genetically susceptible subjects. A longitudinal study that closely follows levels of viral latent and lytic gene expression and cellular changes, in the context of genetic risk alleles will provide an improved understanding of EBV reactivation in SLE and how this reactivation may contribute to autoimmune response.

Mouse models, either humanized or MHV infection of lupus prone mice, may be an alternate approach to decipher the role of EBV. CD34+ hematopoietic stem cells generated *in vitro* from induced pluripotent stem cells (iPSC), which are EBV negative, to reconstitute BLT mice described by Melkus et al. can overcome the effects of prior exposure to EBV in patient cells. The use of iPSC also allows for introducing (or reverting) specific mutations to further clarify the gene/environment interactions, and determining immune dysregulation immediately following EBV infection.

## Author Contributions

All authors contributed to the article and approved the submitted version.

## Funding

This study was supported by the National Institute of Allergy, and Infectious Diseases (R03 AI139975, U19AI082714, U01AI101934), the National Institute of General Medical Sciences (U54GM104938), and the National Institute of Arthritis, Musculoskeletal and Skin Diseases (P30AR073750).

## Conflict of Interest

The authors declare that the research was conducted in the absence of any commercial or financial relationships that could be construed as a potential conflict of interest.
